# Causal associations between fluid intake patterns and dermatitis risk: a Mendelian randomization study

**DOI:** 10.3389/fnut.2024.1416619

**Published:** 2024-08-14

**Authors:** Ruiqi Zeng, Beian Guo, Wanzhe Liao, Kairui Zhuan, Huilan Chen, Zixiang Qin, Junxi Lin, Tingyu Gu, Zhiyi Zhou

**Affiliations:** ^1^Guangzhou Medical University, Guangzhou, China; ^2^Department of Clinical Medicine, The Nanshan College of Guangzhou Medical University, Guangzhou, China; ^3^Department of Clinical Medicine, The Third Clinical School of Guangzhou Medical University, Guangzhou, China; ^4^Department of Baijiu, Sichuan University Jinjiang College, Meishan, China; ^5^Department of Electrical Engineering, Northwest Minzu University, Lanzhou, China; ^6^Department of Biomedical Engineering, Guangdong Medical University, Dongguan, China

**Keywords:** fluids intake, dermatology, atopic dermatitis, contact dermatitis, Mendelian randomization

## Abstract

**Background:**

Dermatitis is one of the most common skin disorders across the world. Atopic dermatitis (AD) and contact dermatitis (CD) are its two primary types. Few studies have focused on the causal relationship between fluid intake and dermatitis. With an Mendelian Randomization (MR), this study investigated the potential causal effects of alcohol, coffee, tea, and water intake on the risk of AD and CD.

**Methods:**

Utilizing genetic variants as instrumental variables (IVs), a two-sample MR analysis was implemented based on data from the UK Biobank and FinnGen r9 consortium. Fluid intake was categorized into alcohol, coffee, tea, and water intake. Causal estimates were analyzed through Inverse Variance Weighted (IVW), MR-Egger, and weighted median methods. Cochran’s Q, MR-Egger intercept, and MR-PRESSO tests were conducted to assess potential heterogeneity and pleiotropy.

**Results:**

Water intake exhibited a significant causal effect on raised CD risk (IVW OR = 2.92, 95% CI: 1.58–5.41, *p* = <0.01). Coffee intake was associated with increased CD risk (IVW OR = 2.16, 95% CI: 1.19–3.91, *p* = 0.01). Conversely, tea intake demonstrated a protective effect on AD risk (IVW OR = 0.71, 95% CI: 0.56–0.91, *p* = <0.01).

**Conclusion:**

This MR study suggests a potential association where water and coffee intake may be linked to an elevated risk of CD, while tea intake may potentially have a mitigating effect on AD risk. Modifying fluid intake patterns could be a targeted approach for dermatitis prevention, emphasizing the need for additional longitudinal studies to validate and expand upon these findings.

## 1 Introduction

Atopic dermatitis (AD) and contact dermatitis (CD) are two primary types of dermatitis, each with distinct characteristics and etiologies.

AD, commonly referred to as atopic eczema, manifests as a persistent inflammatory skin condition characterized by a multifaceted pathophysiology involving both genetic susceptibility and environmental stimuli (1). The prevalence of this chronic ailment is on the rise globally, impacting approximately 20% of children and around 3% of adults in the world (2). Initially categorized as an allergic skin disorder, AD is now recognized as a highly intricate condition exhibiting a diverse range of clinical manifestations (2). It has been demonstrated that the emergence of AD involves the intricate interplay of a variety of conditions. In the genesis of AD, genetic risk factors exert significant effects in affecting both the skin barrier and the immune system. In addition, environmental factors are acknowledged contributors to the development of the condition (2). Factors such as changes in the microbiome and immune dysregulation are also implicated in this multifaceted scenario (3). However, in regards to the etiology of AD, its causes and relevant mechanisms are still not comprehensively understood (4). Previous observational studies have suggested that the risk of autoimmune diseases is higher in patients with AD, especially those affecting the skin and digestive system (5, 6).

CD is one of the prevalent inflammatory skin diseases triggered by contact with exogenous substances or exposure to specific allergens, provoking an immune response and consequently leading to skin inflammation (7). CD can happen at any stage of life. According to previous studies, the incidence of CD ranges from 1.7 to 6.3% (8). Regarding gender and age, CD is found to be more common in women and the elderly (8). Dry air, hotness, liquids like alcohol, and other environmental factors are widely considered to be the common causes of CD. Frequent contact with mild stimuli such as water and some cosmetics over a long period can also lead to CD (9).

Both AD and CD are major types of dermatitis, but they differ in their etiology and pathophysiology. AD is primarily driven by genetic and environmental factors that affect the skin barrier and immune response, often associated with a history of atopy (1). CD, on the other hand, is caused by direct contact with allergens or irritants that provoke an immune response leading to skin inflammation (7). Understanding these differences is crucial for identifying potential causal relationships between various exposures and the risk of dermatitis.

Several studies have explored the connection between fluid intake and the risk of dermatitis. However, their causalities are still elusive. Recent observational studies have presented evidence demonstrating that dermatitis can result from airborne exposure to coffee beans, as well as direct contact with instant coffee and coffee powder (10–12). Hinton AN et al. conducted a review proposing a correlation between alcohol consumption and dermatitis (13). Nonetheless, past research relies on observational epidemiological methodologies, which are susceptible to reverse causation and other confounding, making it difficult to establish clear causal inferences (14). Furthermore, there is a notable absence of studies substantiating the causalities between various fluid intake and the risk of dermatitis, and their causalities remain ambiguous and warrant further investigation.

Mendelian randomization (MR) serves as a method to explore causality by deducing the causal relationship between exposure and outcome through the use of genetic variants as instrumental variables (IVs), which are often represented by single nucleotide polymorphisms (SNPs), operate independently of confounding factors or reverse causality (15). In comparison to observational studies, MR analysis proves advantageous in bypassing confounding variables and mitigating the impact of reverse causation (16, 17). In addition, the latest progress in genome-wide association studies (GWASs) has revealed an abundance of genetic variants strongly linked to complex human diseases and traits, thereby furnishing an extensive pool of potential IVs that enhance the effectiveness of MR analysis (18). In the current research, a two-sample MR analysis was employed to delve into the causal effects of the intake of various fluids—alcohol, coffee, tea, and water—on the risk of both AD and CD.

## 2 Materials and methods

### 2.1 Summary of study design

**Figure 1:**
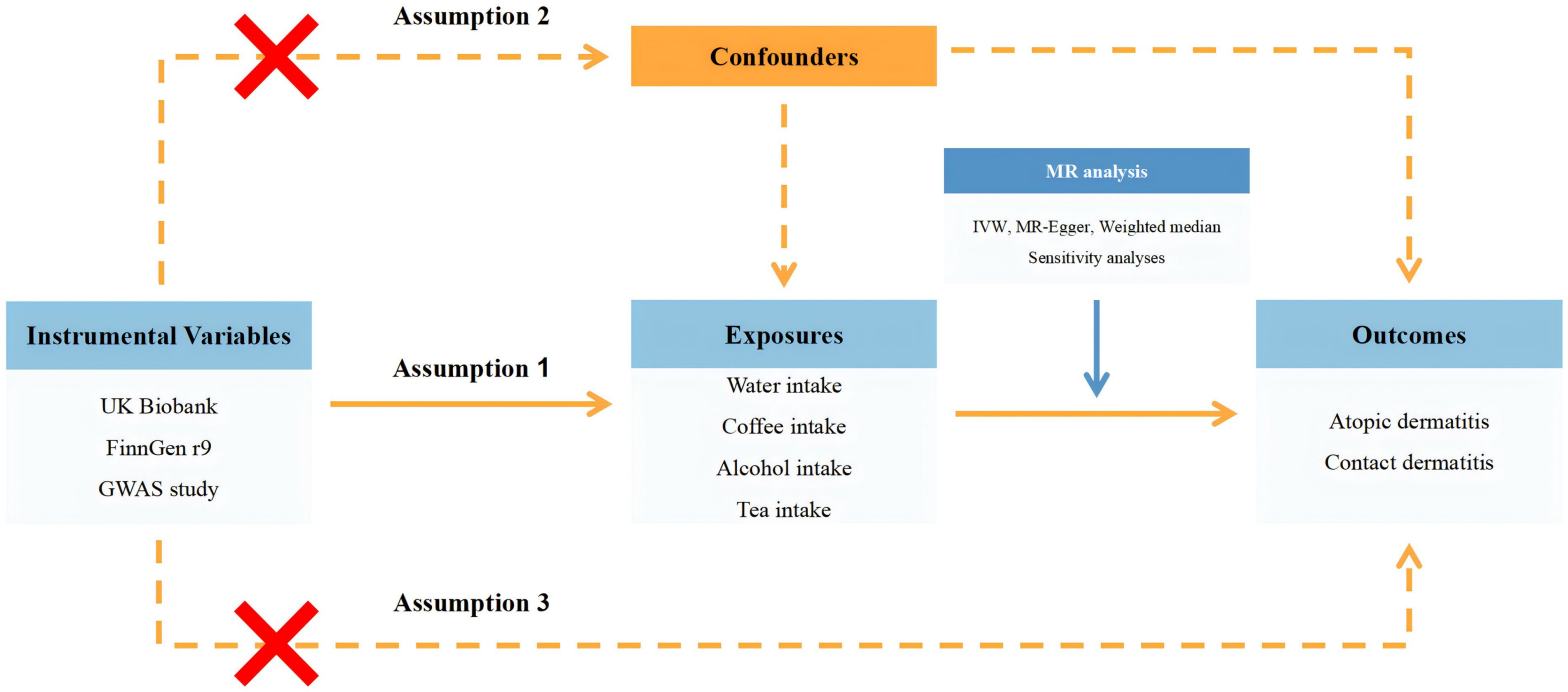
Flow chart of the entire study design. GWAS, genome-wide association study; MR, Mendelian randomization; UVMR, univariable Mendelian randomization; IVW, inverse variance weighted.

The entire study design was summarized in Figure 1 and presented in the form of a flow chart.

### 2.2 Data sources

Summary data of fluids intake and dermatitis utilized in this MR study are described in Table 1. Summary information for fluids intake and dermatitis in this research are shown in Table 1. In this study, fluid intake was considered as the exposure and categorized into alcohol, coffee, tea, and water intake. Summary statistical data of fluid intake were acquired from MRC-IEU analysis of the UK Biobank database (Alcohol intake: sample size = 462,346; Coffee intake: sample size = 428,860; Tea intake: sample size = 447,485; Water intake: sample size = 427,588). Quantitative data of fluid intake from the UK Biobank were used to conduct this study. Fluid intake data were obtained from the UK Biobank.^1^ AD and CD were considered as the outcome, and their summary data were acquired from FinnGen r9 consortium (sample size = 32,457) and a large-scale GWAS published by Sakaue S et al. (sample size = 478,766), respectively (19). Summary data of AD were obtained from the FinnGen consortium.^2^ Phenotypes used in this study were available online at the Integrative Epidemiology Unit (IEU) OpenGWAS Project website.^3^ This study exclusively involved participants of European ancestry, and all summary data are publicly accessible. Table 1 described the detailed information of summary-level data in this MR study.

**Table 1:** Detailed information of summary data in this MR study.

Data source	Phenotype	Sample size	Population
UK Biobank	Water intake	427,588	European
UK Biobank	Coffee intake	428,860	European
UK Biobank	Alcohol intake	462,346	European
UK Biobank	Tea intake	447,485	European
Sakaue S et al.	CD	478,766	European
FinnGen r9	AD	32,457	European

CD, contact dermatitis; AD, atopic dermatitis.

### 2.3 IVs selection

In general, genetic variations served as IVs to elucidate the connection between different fluid intake and the risk of both AD and CD. The selection process was in accordance with three fundamental assumptions within the MR analysis (20, 21). Firstly, IVs should exert a direct and statistically significant influence on fluid intake. Secondly, IVs should solely impact dermatitis through their effects on fluid intake. Thirdly, IVs should be rigorously independent of any potential confounders. Hence, our primary criterion for screening candidate SNPs was to establish *p*-values for their correlation with exposure less than 5e-8, retaining SNPs with r2 < 0.001 and a clump distance less than 10,000 kb for independent loci identification. Additionally, SNPs with minor allele frequencies (MAF) less than 0.01 were excluded due to their typically lower confidence levels. To validate the estimated causal direction, we conducted an MR-Steiger analysis for confirmation. To eliminate SNPs potentially influenced by confounding factors, we referred to each SNP in the PhenoScanner database to ensure that the selected IVs were independent of any potential confounders (22). Phenoscanner served as the tool for identifying factors correlated to the outcome or its confounders, which were usually air pollution, dry air, high temperature, occupational and environmental exposures to metalworking fluids and plants, as well as continuous exposure to mild irritants like cleansing gel (9, 23–25). F-statistic was calculated to detect the existence of weak IVs. Generally, an F-statistic exceeding 10 is considered indicative of weak IV bias. The F-statistics for all SNPs were over 10, suggesting that there was no weak instrumental bias.

### 2.4 MR analysis

Following the retrieval of the selected SNPs, a harmonization process was implemented to align the alleles and effects between the exposure and outcome and exclude all palindromic SNPs. Further statistical analyses for MR were carried out utilizing the screened SNPs. In order to explore the causality between various fluid intakes and the liability of dermatitis, a two-sample MR analysis was conducted through diverse approaches, including the Inverse Variance Weighted (IVW), MR-Egger, and weighted median. These methods produce different assumptions and deal with pleiotropy effects by varied means (20, 26, 27). Odds Ratios (ORs) for the risk of dermatitis were calculated per 1 standard deviation (SD) increase in the fluids intake. When the three essential MR assumptions mentioned earlier are satisfied, the IVW method possesses the highest statistical power and is considered more robust for estimation with heterogeneity among present (27). As a result, the outcomes derived from IVW were deemed the principal part of causal effect evaluation. Based on the fact that the weighted median method operates relying on the assumption that a minimum of half of the IVs are considered valid, and the MR-Egger method yields a causal estimate even if all IVs are invalid, the results obtained from both approaches were utilized to validate the overall direction of the effect. Despite being less efficient, we utilized MR-Egger and weighted median methods for their capacity to deliver more significant results in a wider range of circumstances (20, 26). Scatter plots, funnel plots, and Leave-One-Out analyses were performed on the causality of fluid intake and the liability of dermatitis. After applying the Bonferroni correction, statistical significance was deemed present with a threshold of *p* < 0.025.

### 2.5 Heterogeneity and pleiotropy analyses

We conducted Cochran’s Q test and MR-Egger intercept test to detect any latent heterogeneity and pleiotropy among IVs, with *p* < 0.05 indicating significant heterogeneity and intercepts significantly different from zero suggesting the presence of horizontal pleiotropy, respectively. MR Pleiotropy RESidual Sum and Outlier (MR-PRESSO) method was employed to identify latent outlier IVs and subsequently eliminate them (28). The research process adhered to the guidelines outlined in the STROBE-MR statement (29). All analyses were conducted through TwoSampleMR package (version 0.5.7), and RadialMR (version 1.1) in R (version 4.3.1).

## 3 Results

**Figure 2:**
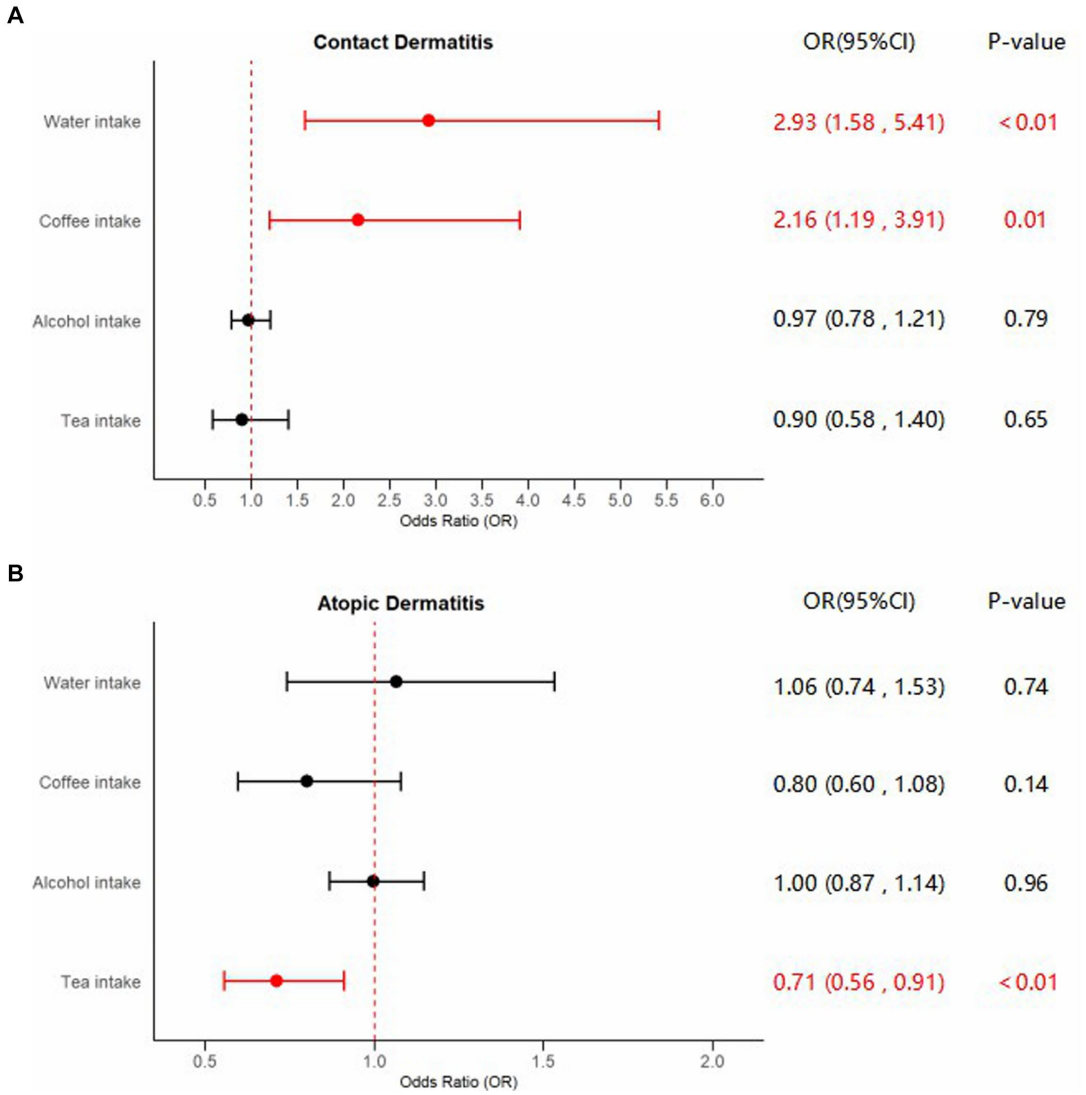
Forest plots of significant estimates of MR analyses. The IVW method was employed to explore the causalities of fluid intake on dermatitis risk. **(A)** MR results of fluid intake on contact dermatitis risk; **(B)** MR results of fluid intake on atopic dermatitis risk. OR, odds ratio; CI, confidence interval; IVW, inverse variance weighted; MR, Mendelian randomization. Statistically significant results are indicated in red, with error bars representing 95% confidence intervals.

Supplementary Table S1 showed Steiger directional tests results. The characteristics of the SNPs employed to estimate the causal effects of liquid intake on the risk of dermatitis were shown in Supplementary Tables S2–S9. Forest plots of significant estimates of MR analyses based on IVW method were displayed in Figure 2.

### 3.1 Causal estimates between fluids intake and the risk of CD

**Figure 3:**
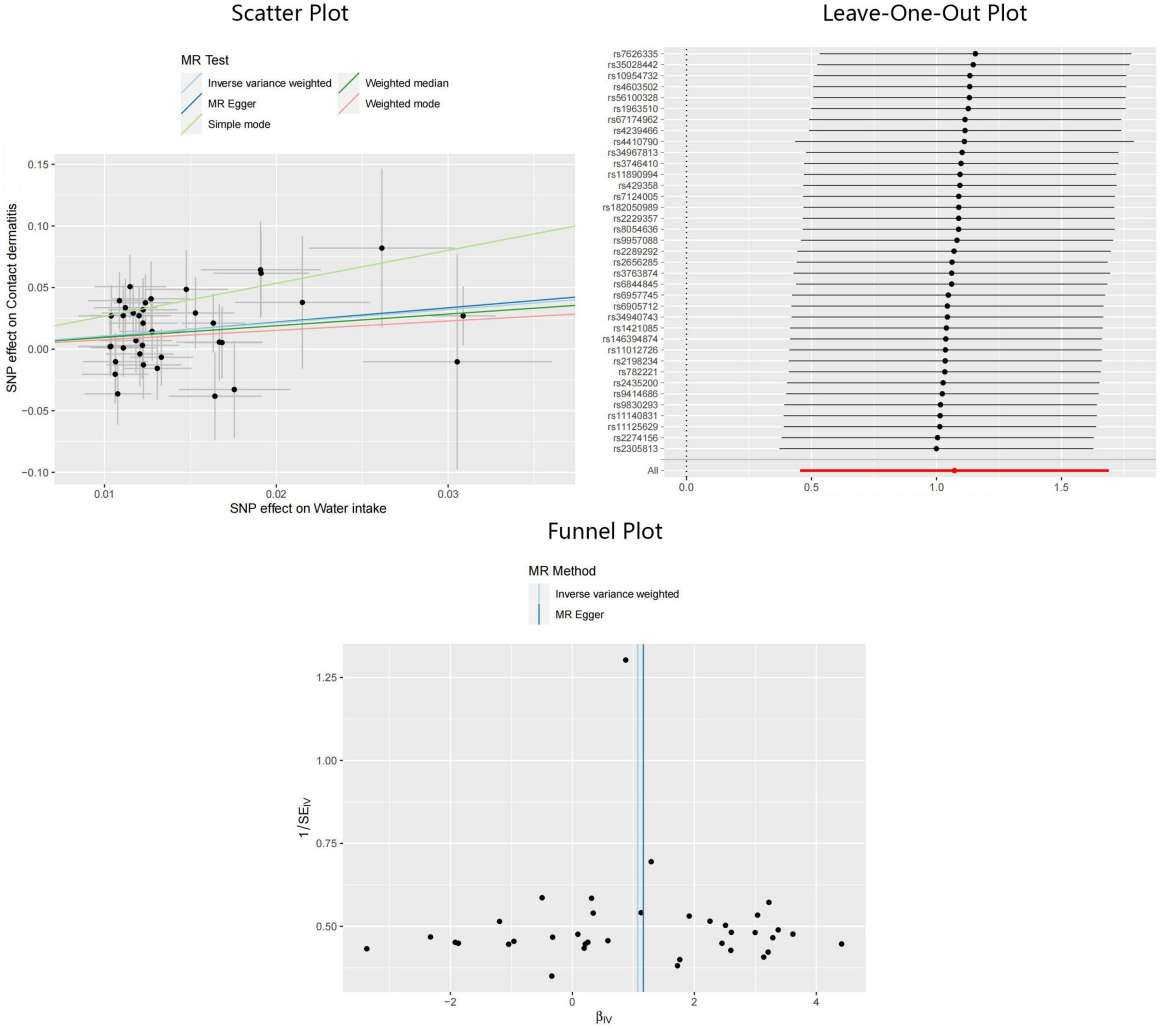
Scatter plot, Leave-One-Out plot, and funnel plot for the estimated causal effect of water intake on the risk of CD. SNP, single nucleotide polymorphism; CD, contact dermatitis; IVW, inverse variance weighted.

Compelling evidence supporting the causal effect between water intake and CD was uncovered, employing the IVW model: odds ratio (OR) = 2.92, 95% confidence interval (CI): 1.58–5.41, *p* < 0.01. Estimates from MR-Egger and weighted median models provided identical direction with IVW, without robust evidence for statistical significance: OR = 3.20, 95%CI: 0.41–24.71, *p* = 0.27 for MR-Egger, and OR = 2.58, 95%CI: 0.96–6.98, *p* = 0.06 for weighted median. No potential heterogeneity and pleiotropy were found through Cochran’s Q test (P_MR-Egger_ = 0.75, P_IVW_ = 0.78) and MR-Egger intercept test (*p* = 0.93). The MR-PRESSO test was implemented to further validate the results (*p* = 0.81). The corrected IVW estimator generated by MR-PRESSO was consistent with the results of IVW method, confirming the robustness of our results. All Steiger directional tests indicated no reversal causality. Scatter plots, Leave-One-Out plots, and funnel plots of the estimated causality of water intake on CD was integrated and displayed in Figure 3.

**Figure 4:**
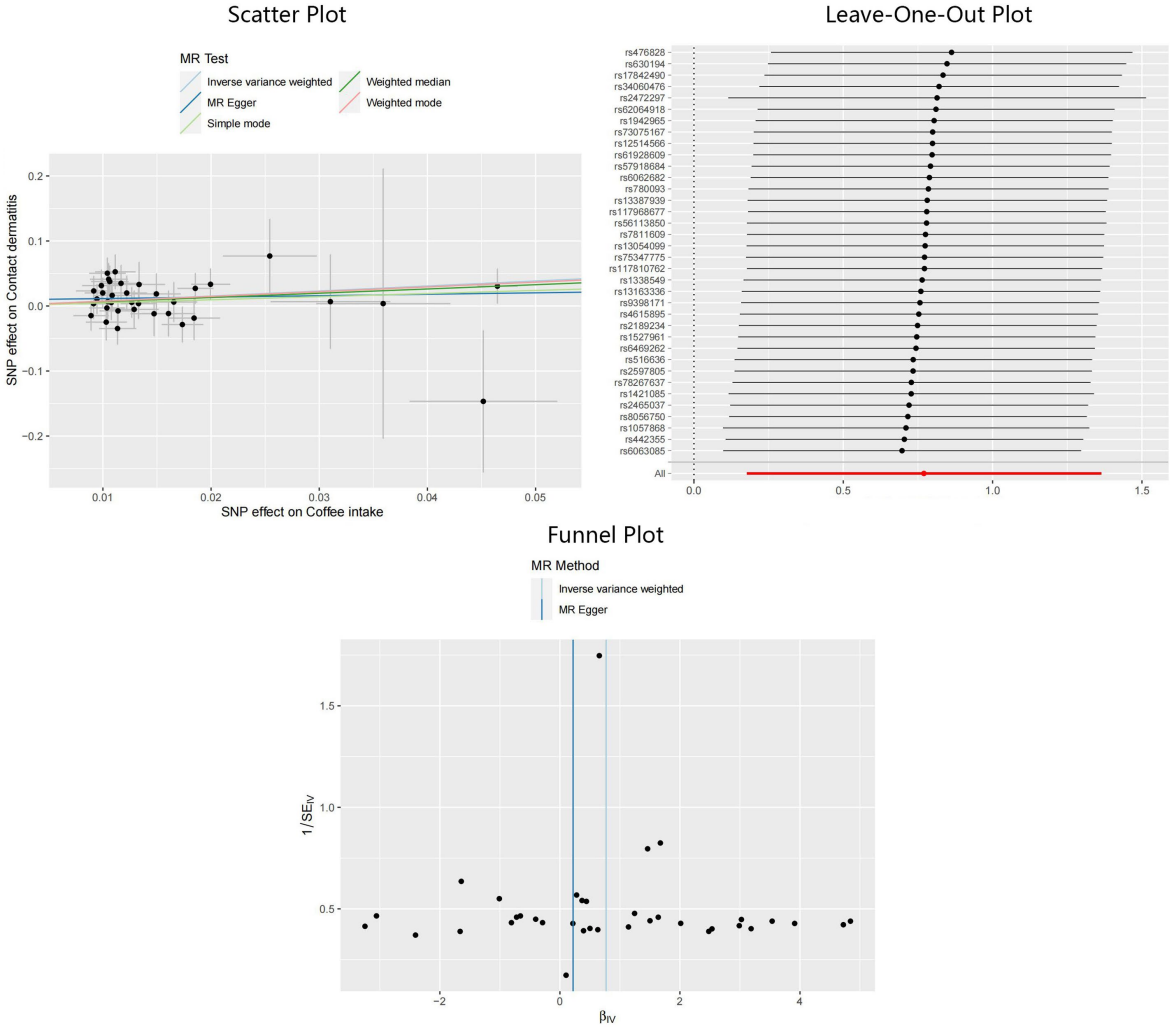
Scatter plot, Leave-One-Out plot, and funnel plot for the estimated causal effect of coffee intake on the risk of CD. SNP, single nucleotide polymorphism; CD, contact dermatitis; IVW, inverse variance weighted.

In addition, applying the IVW method, we also identified significant results indicating the causality of coffee intake on CD: OR = 2.16, 95%CI: 1.19–3.91, *p* = 0.01. Although not statistically significant, outcomes from the MR-Egger and weighted median methods indicated effect directions identical to that derived from the IVW method: OR = 1.25, 95%CI: 0.34–4.61, *p* = 0.74 for MR-Egger, and OR = 1.93, 95%CI: 0.73–5.11, *p* = 0.19 for weighted median. Scant evidence supported the existence of any potential heterogeneity and pleiotropy according to the results of Cochran’s Q test (P_MR-Egger_ = 0.78, P_IVW_ = 0.79), MR-Egger intercept test (*p* = 0.36), and MR-PRESSO test (*p* = 0.81). The IVW estimator corrected by MR-PRESSO aligned with the results of the IVW method, reinforcing the robustness of our findings. Scatter plots, Leave-One-Out plots, and funnel plots of the estimated causality of coffee intake on CD was integrated and displayed in Figure 4. On the contrary, concerning the causalities of alcohol and tea intake on the liability of CD, limited evidence suggested a statistically significant association. All Steiger directional tests indicated no reversal causality. Scatter plots, Leave-One-Out plots, and funnel plots of the estimated causalities of alcohol and tea intake on CD were integrated and displayed in Supplementary Figures S1, S2.

### 3.2 Causal estimates between fluids intake and the risk of AD

**Figure 5:**
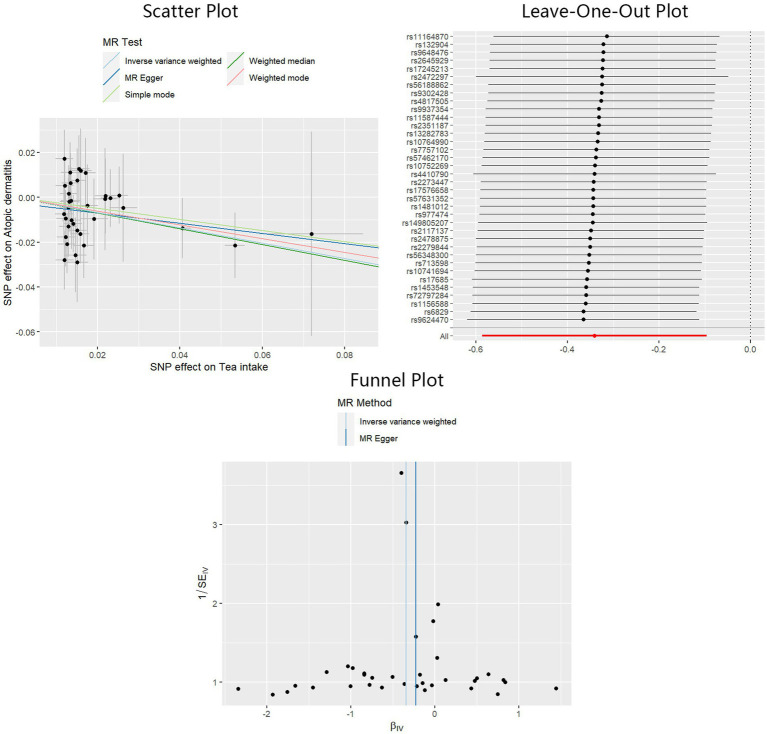
Scatter plot, Leave-One-Out plot, and funnel plot for the estimated causal effect of tea intake on the risk of AD. SNP, single nucleotide polymorphism; AD, atopic dermatitis; IVW, inverse variance weighted.

A significant causal effect of tea intake on the risk of AD was revealed in the IVW analysis: OR = 0.71, 95%CI: 0.56–0.91, *p* < 0.01. The causal effect consistently exhibited the same direction, although statistical significance was not achieved in the MR-Egger and weighted median methods: OR = 0.80, 95%CI: 0.47–1.36, *p* = 0.41 for MR-Egger, and OR = 0.70, 95%CI: 0.49–1.00, *p* = 0.05 for weighted median. Sensitivity analyses indicated the absence of significant heterogeneity or horizontal pleiotropy, as inferred from the results of Cochran’s Q test (P_MR-Egger_ = 0.90, P_IVW_ = 0.91), MR-Egger intercept test (*p* = 0.64), and MR-PRESSO test (*p* = 0.93). The adjusted IVW estimator produced by MR-PRESSO was consistent with the results obtained using the IVW method, ensuring the robustness of our findings. Scatter plots, Leave-One-Out plots, and funnel plots of the estimated causality of tea intake on AD was integrated and displayed in Figure 5. No significant causality of the intake of water, coffee, and alcohol on the risk of AD was found. All Steiger directional tests indicated no reversal causality. Scatter plots, Leave-One-Out plots, and funnel plots of the estimated causalities of water, coffee, and alcohol intake on AD were integrated and displayed in Supplementary Figures S3–S5.

## 4 Discussion

As far as our knowledge extends, the causal relationship between fluid intake and the risk of dermatitis has yet to be elucidated. Employing a comprehensive MR analysis through various approaches, this study acts as a pioneer endeavor to explore the latent causalities between the intake of different fluids intake and the risk of dermatitis, which was subdivided into CD and AD.

AD is a common inflammatory skin disorder featuring recurrent eczematous lesions and intense pruritus. This condition influences individuals of all ages and ethnicities, exerting a significant impact on both patients and their families. Furthermore, it stands as the primary contributor to the worldwide burden of dermatological diseases. AD is linked to an elevated risk of various comorbidities, such as food allergy, asthma, and mental health disorders (3). CD, on the other hand, represents a type IV delayed hypersensitivity reaction against a non-infectious antigen orchestrated by Th1 cells and CD8+ cytotoxic T cells. These inappropriate immune responses involve cytokines and phagocytes, encompassing an initial sensitization stage that primes the immune system against the allergen. Subsequently, an elicitation phase ensues, characterized by itching and redness (30).

With the aim of mitigating the impact of confounding risk factors and potential reverse causation, we conducted a two-sample MR analysis on the causalities between alcohol, coffee, tea, water intake and the risk of CD and AD. This MR study demonstrates that water and coffee intake can increase the risk of CD. In comparison, tea intake exerts a protective influence on AD. There was no evidence supporting a causality between alcohol intake and the risk of CD and AD.

Previous investigations have also explored the associations between water exposure and dermatitis. Long-standing beliefs connect CD with exposure to solvents, oils, and surfactants such as soap and detergents (31). Fujiwara N et al. demonstrated that the absorption of laurate, a key constituent of soap, was heightened in the presence of calcium, the primary cationic element in natural water systems, commonly referred to as water hardness (32). A clinical trial by Warren R et al. revealed that water hardness, also known as calcium in water, could contribute to CD susceptibility and affect the severity of symptoms. Two types of effects of water hardness on the biological response of skin to surfactants were responsible: a direct effect of calcium on the skin barrier and an indirect effect on the interaction between surfactants and calcium (33). Bains SN et al. reported in a review that repetitive exposure to mild irritants, including soap and water, significantly increased the risk of CD (34). A retrospective and cross-sectional study by Lee et al. demonstrated that skin transepidermal water loss could act as a biomarker for the intensity of itch caused by AD, though not for disease severity (35). It is noteworthy that certain occupations, such as medical personnel, hairdressers, and food workers, increase the risk of CD due to repeated contact with substances, including water, detergents, and other chemicals, aligning with previous findings (36). Water exposure exerts complicated effects on the skin, potentially inducing significant alternations such as epidermal thickening, dilation of the intercellular space, and changes in Langerhans cells and mononuclear cells in the epidermis (37). Warner et al. delved into a mechanism through which the intercellular lipid structure was impaired by water and the breakdown of corneodesmosomes, resulting in enhanced skin permeability, similar to the effects observed with surfactant solutions (37, 38). Water intake might have some of the same effects, but this requires further research for confirmation.

Current research indicates that the consumption of coffee contributes to the aggravation of CD, primarily owing to its diverse chemical components, such as nickel and chromium (39). Additionally, multiple previous studies have consistently shown the promotional impact of nickel on CD (40–42). Silverberg NB et al.’s study indicated that skin exposure to nickel had the potential to induce a type-IV cutaneous hypersensitivity reaction in two steps. In the initial stage, skin dendritic cells presented nickel as an allergen to Th1 and Th17 cells, leading to the formation of a set of memory T cells that recognized nickel. With repeated exposure to nickel, the process of allergy elicitation was activated, ultimately culminating in the development of CD (41). Furthermore, Jensen CS et al. proposed that oral nickel exposure induced cutaneous nickel-allergic reactions in a dose-dependent manner, echoing our findings that coffee intake could increase the risk of CD (40). A transcriptome analysis by Lukas Wisgrill et al. revealed that late-phase nickel challenge had the potential to induce notable alterations in leukocyte composition, encompassing the recruitment and activation of NK cells, polarization of macrophages, and modulation of T-cell immunity, with substantial upregulation of MMP12 and SOCS3 (43). Compared to experimental irritant skin responses induced by sodium lauryl sulfate, nickel-induced allergic skin responses uniquely exhibited infiltration of NK cells and activation of cytotoxic pathways (43). In an animal study by Vibha Dube et al., a remarkable increase in leukocyte response was noted in experimental CD mice induced by chromate injection. This highlighted chromium’s potential to induce CD through a delayed hypersensitivity reaction mediated by sensitized cells. The release of pharmacological mediators following the degranulation of these cells exerted a direct and crucial influence on the pathophysiology of CD (44). Buters J et al. delved into more specific mechanisms, providing additional insights. Their studies suggested that chromium-induced cytotoxicity and hypersensitivity predominantly involved the activation of the NLRP3 inflammasome, resulting in the release of interleukin-1β (IL-1β). Although chromium in the human body primarily exists in the trivalent form, it can undergo oxidation and transform into hexavalent chromium under specific conditions, serving as the primary culprit exacerbating the process of CD. The application of antioxidants may exert protective effects on CD, as NLRP3 activation depends on the accumulation of reactive oxygen species induced by hexavalent chromium (45).

The consumption of tea has long been acknowledged for its positive impact on human health. Antiinflammation, one of its healthy beneficial effects, has been reported in previous studies on diverse kinds of tea, including green tea, oolong tea, and black tea (46, 47). According to a review by Zink A et al., numerous studies have presented a promising perspective regarding the utilization of green tea as a viable alternative treatment for chronic, infectious, inflammatory, and hair disorders. Additionally, it is regarded as a preventive measure, acting against both skin aging and skin cancer (48). Green tea consists of diverse chemical components, including catechins, caffeine, organic acids, polyphenols, and theanine. EGCG, identified as the primary and most bioactive polyphenol in green tea, enhances the expression of the antioxidative enzyme HO-1, regulates MAP kinases, inhibits the proteasome, and indirectly diminishes TLR signaling, thus exerting antioxidative, antitumor, and anti-inflammatory effects (48, 49). Mouse studies have demonstrated that theaflavins, a substantial component of black tea, play a notable role in exerting antiallergic effects, potentially by inhibiting cytokine production from Th2 cells and suppressing oxidative stress induced by active oxygen species (46). Another study using animal models disclosed that the administration of tea, encompassing green, black, and oolong tea, effectively suppressed type I and type IV allergic reactions (50). This finding was clinically tested and validated in a subsequent study, affirming the effectiveness of oolong tea in AD, presumably attributed to the antiallergic properties of tea polyphenols (51). In a clinical context, routine bath therapy utilizing green tea extracts has demonstrated substantial improvement in AD and has been suggested as an effective alternative AD treatment. Evaluated by the Scoring Atopic Dermatitis Index, patients treated with green tea extracts made a good recovery of their AD (52). These studies all supported our findings. Tea intake might reduce the risk of AD by influencing anti-inflammatory and antioxidant effects.

We admit that this MR study possessed some advantages. First, MR provides us with the ability to identify causality by simulating randomized control trials within observational settings. Besides, MR circumvents the need for significant time and financial investments while maintaining a high level of confidence. Second, MR relies on genetic phenotypes assigned at conception to predict causation, making it more resilient to environmental influences and other factors that may compromise correct causal predictions. In contrast to traditional observational studies, MR’s reliance on genetically potent instrumental variables helps prevent reverse causal effects. Third, this research was based on data from the most recent databases, including the UK Biobank and FinnGen r9, along with a large-scale GWAS. The extensive sample size and utilization of the latest databases enhance the study’s reliability and persuasiveness. Fourth, it is the first study employing MR analysis to scrutinize the causal relationship between diverse fluid intakes and the susceptibility to dermatitis, including both AD and CD. Given the global ubiquity of dermatitis and the widespread consumption of alcohol, coffee, tea, and water, unraveling the causative links between fluid intake and dermatitis holds instructive value. These findings not only offer novel perspectives for clinical trials but also possess the potential to shape public health policies aimed at dermatitis prevention and treatment.

However, there were also several limitations. Firstly, the study participants were confined to individuals of European descent, raising questions about the generalizability of the findings to diverse global populations. In addition, we recognize the heterogeneity in the populations studied. While the UK Biobank includes a diverse European population, the Finnish population in the FinnGen cohort is more homogeneous and distinct. Beverage consumption patterns also differ significantly between these populations. These differences could impact the observed associations between fluid intake and dermatitis risk. Secondly, this study concentrated on four widely consumed fluids—alcohol, coffee, tea, and water—in relation to their influence on dermatitis (AD and CD). Nevertheless, it overlooked additional fluids commonly consumed in daily life and failed to address other types of dermatitis, such as fruit juices, carbonated drinks, irritant dermatitis, and stasis dermatitis. Simultaneously, the paper broadly explores fluid intake without delving into their nuanced classifications or considering varying states, concentrations, or temperatures, potentially introducing bias into the analysis. Third, the exploration of fluid intake is confined to oral consumption, neglecting other pathways, such as absorption through skin and mucosa. This limitation hinders a comprehensive understanding of human liquid intake patterns. Furthermore, it is important to acknowledge the limitations associated with the behavioral data on food and beverage intake from the UK Biobank. Despite the fact that the UK Biobank’s data on food intake has been sufficiently validated, reports of the participants may still vary to a certain extent over time due to social desirability bias and changes in dietary habits, posing challenges to the validity of long-term dietary assessments. This variability in self-reported intake introduces potential biases and affects the reliability of our findings, and the inherent biases in behavioral data thereby caused, need to be considered when interpreting the results of our study. Finally, the study, while examining the causal effects of fluid intake on dermatitis, did not explore the specific mechanisms underlying these effects. Additional research is warranted to encompass a more diverse population, a wider array of fluids and dermatitis types, varied methods of fluid intake, and a detailed exploration of the mechanisms underlying the impact of fluid intake on dermatitis.

## 5 Conclusion

In summary, this study suggests a potential association between water and coffee intake with an increased risk of CD, while indicating that tea intake might have a mitigating effect on AD risk. This emphasizes the significance of modifying fluid intake patterns to enhance cutaneous health. For further validation of these discoveries and to achieve a thorough comprehension of the connection between fluid intake and dermatitis, additional research and longitudinal studies are imperative.

## Data availability statement

Publicly available datasets were analyzed in this study. This data can be found here: https://www.ebi.ac.uk/gwas/.

## Author contributions

RZ: Conceptualization, Data curation, Formal analysis, Investigation, Methodology, Visualization, Writing – original draft, Writing – review & editing. BG: Conceptualization, Data curation, Writing – original draft, Writing – review & editing. WL: Data curation, Formal analysis, Software, Writing – original draft. KZ: Data curation, Writing – original draft. HC: Data curation, Writing – original draft. ZQ: Data curation, Writing – original draft. JL: Data curation, Writing – original draft. TG: Data curation, Writing – original draft. ZZ: Conceptualization, project administration, supervision, validation, writing – review & editing.

## Footnotes

^1^ https://www.ukbiobank.ac.uk/

^2^ https://finngen.gitbook.io/documentation

^3^ https://gwas.mrcieu.ac.uk
