# Supplementary material for: Causal associations between fluid intake patterns and dermatitis risk: a Mendelian randomization study
Source: Front Nutr. 2024 Aug 14;11:1416619. doi: 10.3389/fnut.2024.1416619 (PMC11349695; doi:10.3389/fnut.2024.1416619)

Supplementary Figures

Supplementary Fig. 1 Scatter plot, Leave-One-Out plot, and funnel plot for the estimated causal effect of alcohol intake on the risk of CD. SNP, single nucleotide polymorphism; CD, contact dermatitis; IVW, inverse variance weighted.

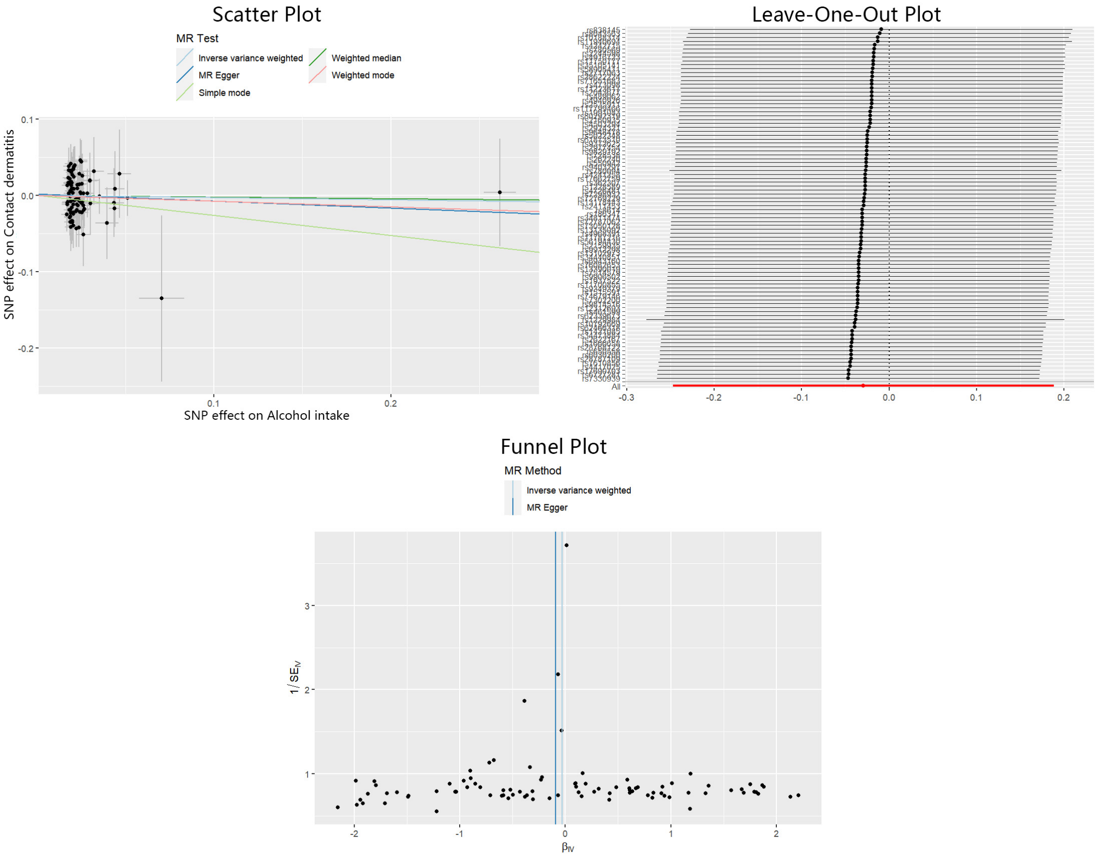

Supplementary Fig. 2 Scatter plot, Leave-One-Out plot, and funnel plot for the estimated causal effect of tea intake on the risk of CD. SNP, single nucleotide polymorphism; CD, contact dermatitis; IVW, inverse variance weighted.

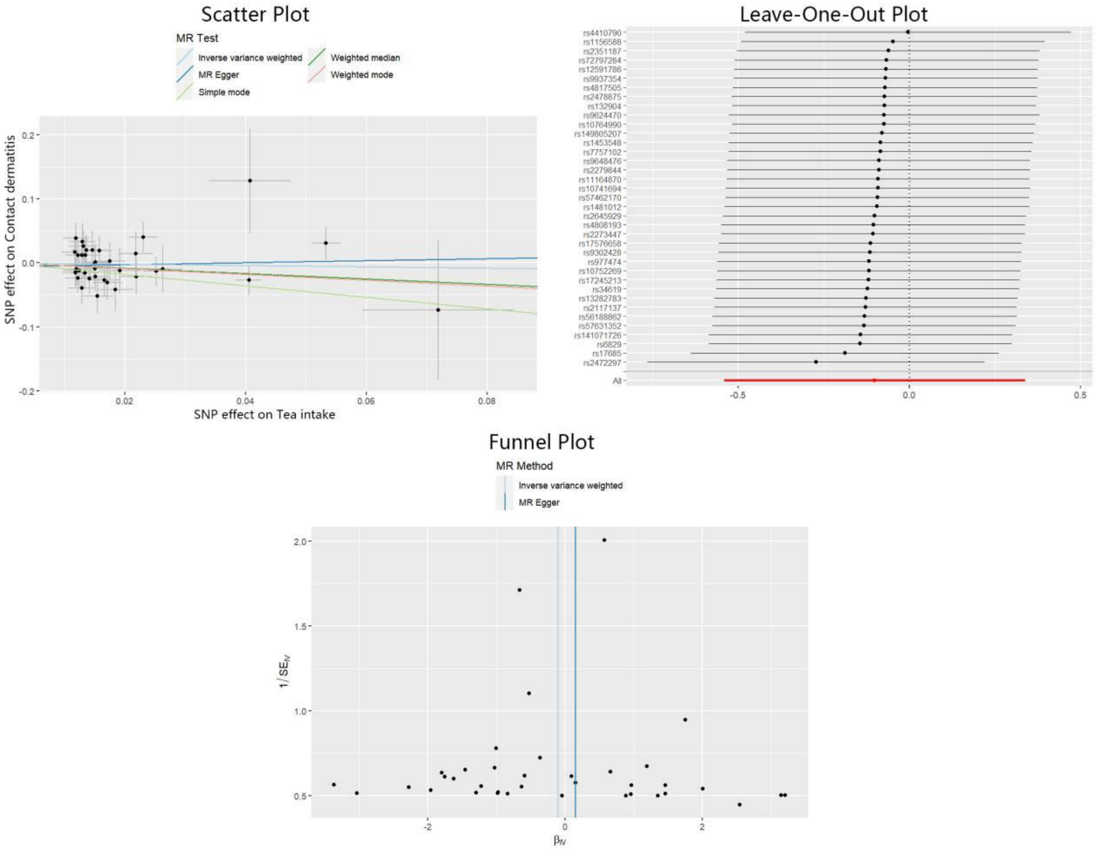

Supplementary Fig. 3 Scatter plot, Leave-One-Out plot, and funnel plot for the estimated causal effect of water intake on the risk of AD. SNP, single nucleotide polymorphism; AD, atopic dermatitis; IVW, inverse variance weighted.

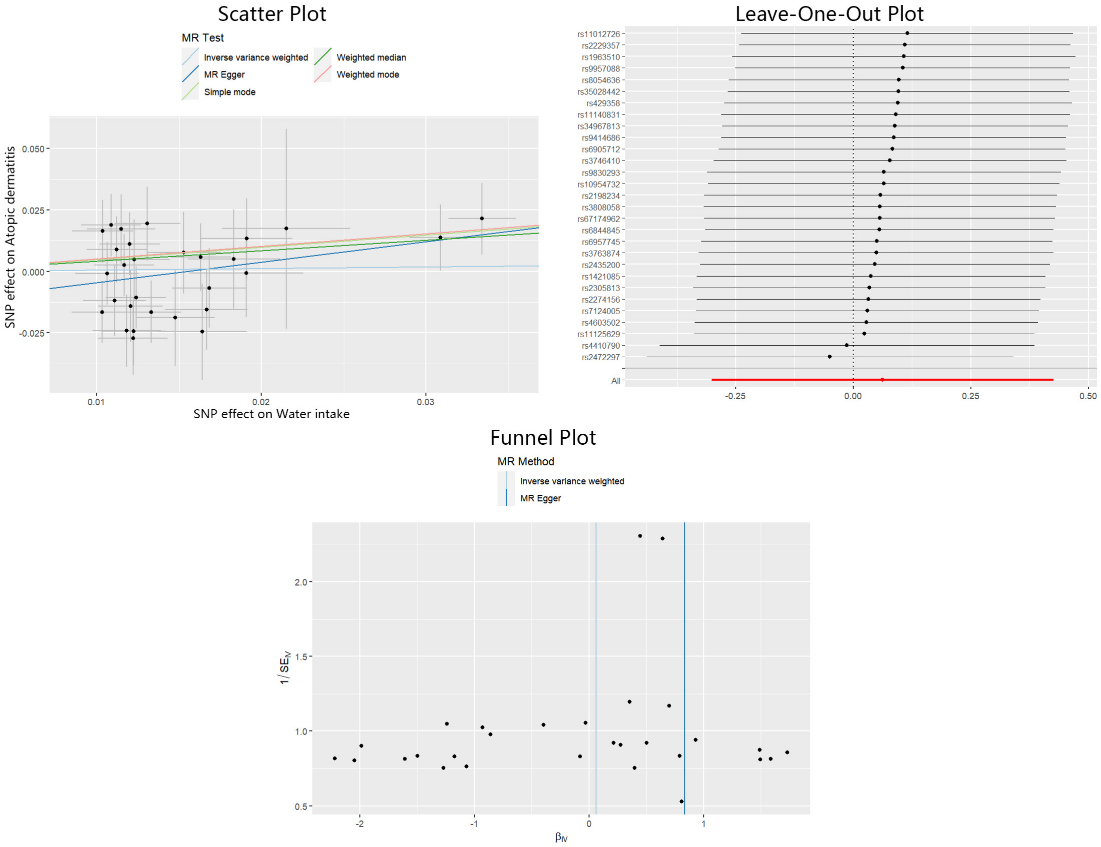

Supplementary Fig. 4 Scatter plot, Leave-One-Out plot, and funnel plot for the estimated causal effect of coffee intake on the risk of AD. SNP, single nucleotide polymorphism; AD, atopic dermatitis; IVW, inverse variance weighted.

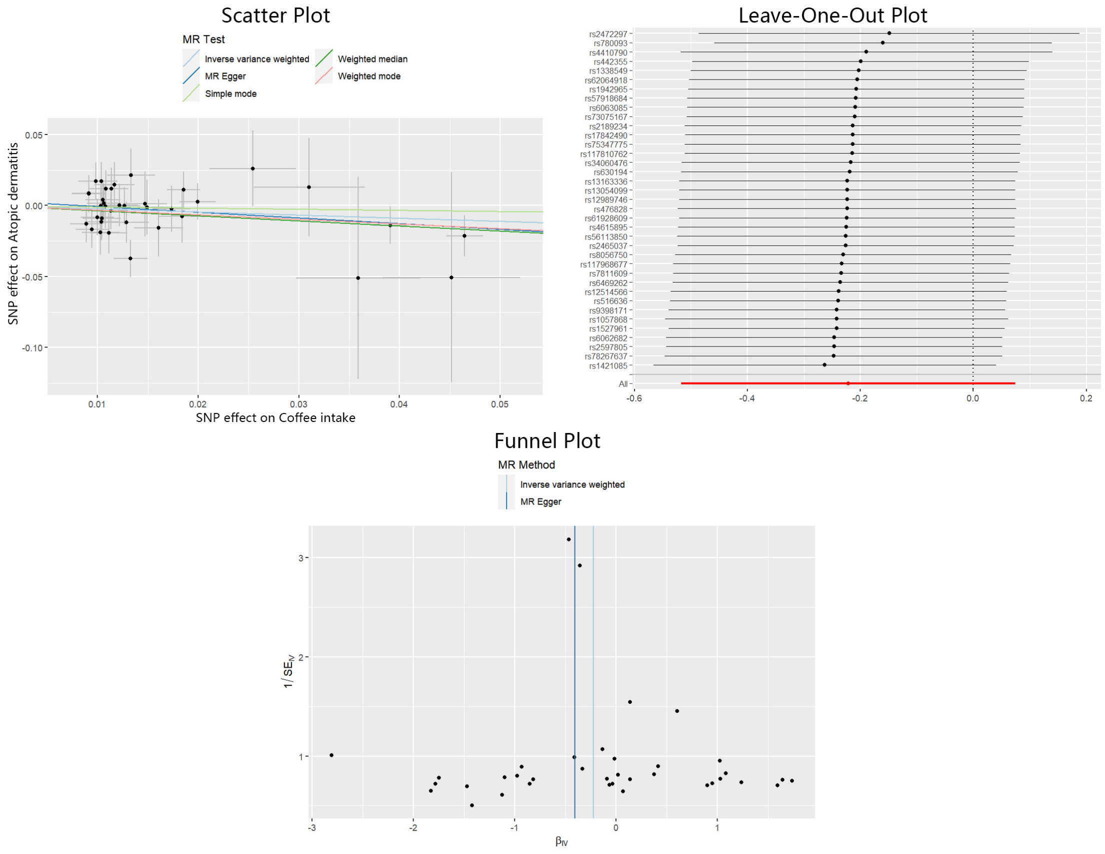

Supplementary Fig. 5 Scatter plot, Leave-One-Out plot, and funnel plot for the estimated causal effect of alcohol intake on the risk of AD. SNP, single nucleotide polymorphism; AD, atopic dermatitis; IVW, inverse variance weighted.

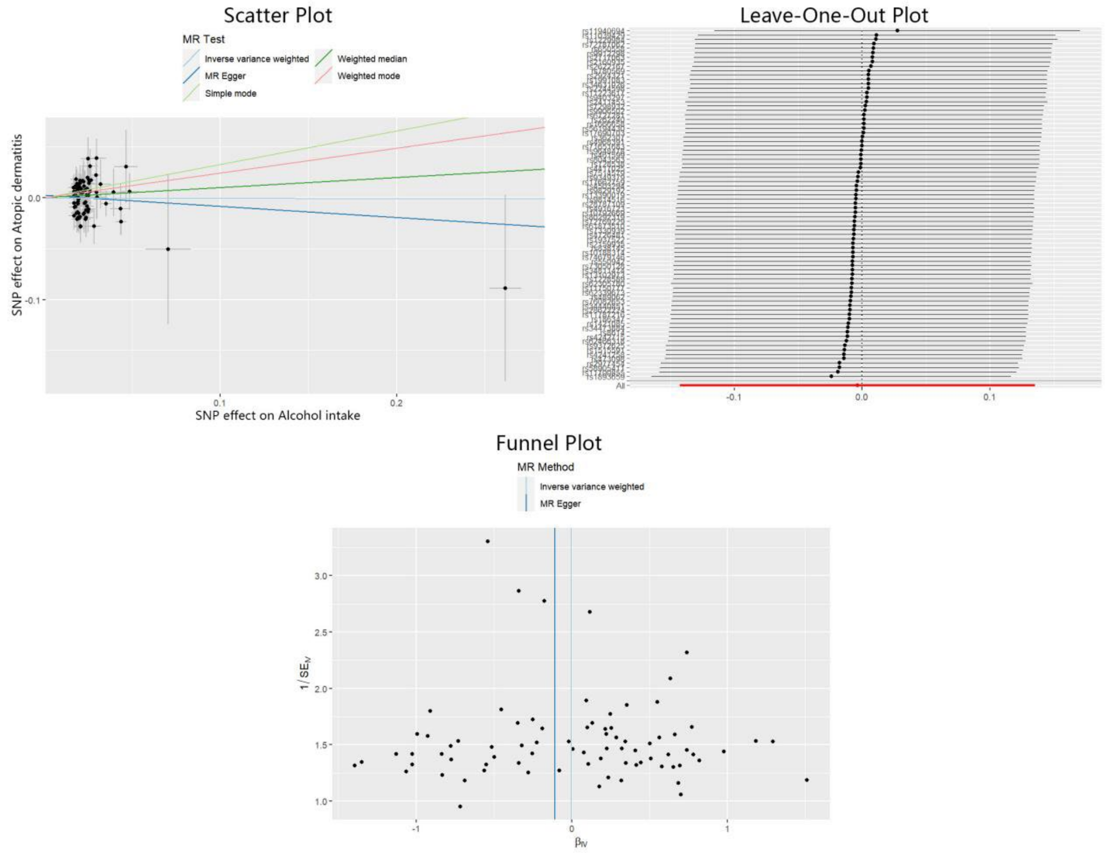

Supplement: Supplementary file 2 [file Image_1.pdf]
